# Characterization of the complete mitochondrial genome of *Diodon hystrix* with phylogenetic consideration

**DOI:** 10.1080/23802359.2017.1331320

**Published:** 2017-05-30

**Authors:** Hai Huang, Hong Guang, Jun Ma, Yan Chen, Huapu Chen

**Affiliations:** a Hainan Key Laboratory for Conservation and Utilization of Tropical Marine Fishery Resources, Sanya, Hainan, China;; b College of Life Science and Ecology, Hainan Tropical Ocean University, Sanya, China;; c Fisheries College, Guangdong Ocean University, Zhanjiang, Guangdong, China

**Keywords:** Mitochondrial genome, *diodon hystrix*, phylogenetic

## Abstract

In this study, the complete mitochondrial genome of *Diodon hystrix* was sequenced and analyzed. The mitochondrial genome is 16,512 bp long and consists of 13 protein-coding genes, two rRNA genes, 22 tRNA genes and a control region. The gene order and composition of *D. hystrix* mitochondrial genome was similar to that of most other vertebrates. The nucleotide composition of the light strand in descending order is 28.69% of A, 31.64% of C, 23.66% of T and 16% of G. The NADH dehydrogenase subunit 6 (ND6) and 8 tRNA genes were localized in the light strand, and all other mitochondrial genes were encoded on the heavy strand. The phylogenetic analysis by maximum-likelihood (ML) method revealed that *D. hystrix* has the closer relationship to the *Diodon holocanthus.*

The *Diodon hystrix* is a commercially important fish species mainly distributed in the Indo-West Pacific region (Soto et al. 2013). With the aim of achieving to find new DNA markers for the studies on population genetics of *D*. *hystrix*, we sequenced the complete mitochondrial genome of *D. hystrix* using the next-generation sequencing (NGS) technique (Chen et al. 2015). *D. hystrix* was obtained in the South China Sea (N18°12′45.00″ E109°19′45.68″) at 5 June 2016 and the muscle of specimen was preserved in 95% ethanol until DNA isolation. The total genomic DNA was extracted by using salting-out procedure (Howe et al. 1997; Chen et al. 2016), and stored in Hainan Tropical Ocean University (Sanya, China) for the subsequent analysis.

The complete mitochondrial genome of *D. hystrix* (Genbank accession number KY677758) is 16,512 bp in length, consisting of 13 protein-coding genes, two ribosomal RNA genes (12S rRNA and 16S rRNA), 22 transfer RNA genes (tRNA) and one control region, which is similar with typical vertebrates (Wang et al. 2008). Most of the genes are encoded on the heavy strand, with only the NADH dehydrogenase subunit 6 (ND6) and 8 tRNA genes [Gln, Ala, Asn, Cys, Try, Glu, Pro, Ser (GCT)] encoded on the light strand. Overall nucleotide compositions of the light strand are 28.69% of A, 31.64% of C, 23.66% of T and 16% of G. However, the most representative base was G and the bias against C was observed, which is different from the base compositions of mitochondrial genome of other teleosts.

There were two types of start codons and four types of stop codons. The two types of start codons were; ATA (ND1, ND5) and ATG of the other 11 genes. Four other types of stop codons are; TAA (ND1, ATP8, ATP6, COX3, ND4L, ND5), TAG (ND2, ND3), AGA (COX1, COX2, CYTB) and AGG (ND4, ND6). The 12S and 16S rRNA genes are localized between the tRNA-Phe (GAA) and tRNA-Leu (TAA) genes. The 22 tRNA genes vary from 67 to 75 bp in length. All these could be folded into the typical cloverleaf secondary structure although numerous non-complementary and T–G base pairs exist in the stem regions. The control region was 849 bp in length, localized between tRNA-Pro (TGG) and tRNA-Phe (GAA) genes. The nucleotide compositions of the control region were 32.08% of A, 20.45% of C, 16.57% of G and 30.90% of T.

The phylogenetic tree for *D. hystrix* was constructed with the complete mtDNA sequences from 13 species of *Tetraodontiformes* by using the maximum-likelihood (ML) methods (Xie et al. 2015; Chen et al. 2016). As shown in Figure 1, the *D. hystrix* was close to *Diodon holocanthus*. Thus, this result supported the monophyly of *D. hystrix*.

**Figure 1.**
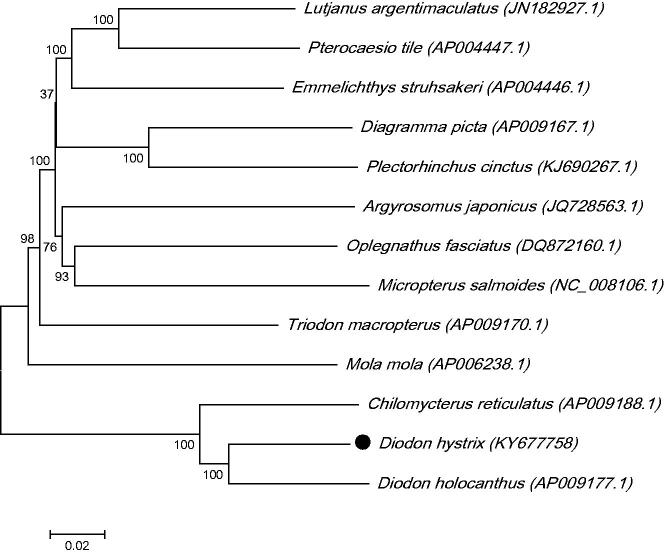
The ML phylogenetic tree of *Tetraodontiformes*. Numbers on each node are bootstrap values of 100 replicates.

## References

[CIT0001] Chen H, Che Z, Li J, Dai M, Xiang L, Deng S, Zhu C, Huang H, Li G. 2015 Illumina next-generation sequencing reveals the complete mitochondrial genome of *Psenopsis anomala* (Perciformes: Centrolophidae) with phylogenetic consideration. Mitochondrial DNA A DNA Mapp Seq Anal. 27:3428–3429. 25974666 10.3109/19401736.2015.1022733

[CIT0002] Chen H, Deng S, Yang H, Ma X, Zhu C, Huang H, Li G. 2016 Characterization of the complete mitochondrial genome of *Priacanthus tayenus* (Perciformes: Priacanthidae) with phylogenetic consideration. Mitochondrial DNA. 1:243–244. 10.1080/23802359.2016.1156493 PMC7871866 33644351

[CIT0003] Howe JR, Klimstra DS, Cordon-Cardo C. 1997 DNA extraction from paraffin-embedded tissues using a salting-out procedure: A reliable method for PCR amplification of archival material. Histol Histopathol. 12:595–601. 9225139

[CIT0004] Soto E, Bogdanovic L, Krecek RC, Jovonovich JA, Arauz M, Overstreet RM. 2013 Parasitosis of Metabronema sp. (Nematoda: Cystidicolidae) in Caribbean porcupinefish, Diodon hystrix (L.). J Fish Dis. 36:1031–1034. 23534535 10.1111/jfd.12109

[CIT0005] Xie Z, Li S, Yao M, Lu D, Li Z, Zhang Y, Lin H. 2015 The complete mitochondrial genome of the *Trachinotus ovatus* (Teleostei, Carangidae). Mitochondrial DNA. 26:644–646. 24090004 10.3109/19401736.2013.836516

